# Reviewer acknowledgement 2014

**DOI:** 10.1186/s40697-015-0036-y

**Published:** 2015-02-10

**Authors:** Adeera Levin, Catherine M Clase, Manish M Sood

**Affiliations:** University of British Columbia, Columbia, Vancouver Canada; McMaster University, Hamilton, Canada; University of Ottawa, Ottawa, Canada

## Abstract

The Editors of *Canadian Journal of Kidney Health and Disease* would like to thank all our reviewers who have contributed to the journal in Volume 1 (2014).

**Stacy Ackroyd-Stolarz**

Canada

**Adebowale Ademola**

Nigeria

**Sofia Ahmed**

Canada

**Sean Barbour**

Canada

**Joanne Bargman**

Canada

**Brendan Barrett**

Canada

**Adam Bass**

Canada

**Monica Beaulieu**

Canada

**Mark Benaroia**

Canada

**Hallgrimur Benediktsson**

Canada

**Martin Bitzan**

Canada

**Josee Bouchard**

USA

**Maxime Bouchard**

Canada

**Darren Bridgewater**

Canada

**Scott Brimble**

Canada

**Dylan Burger**

Canada

**Héloise Cardinal**

Canada

**Fergus Caskey**

United Kingdom

**William Clark**

Canada

**Gabriel Coll-de-Tuero**

Spain

**Jose Cote**

Canada

**Bernard Dickens**

Canada

**Todd Fairhead**

Canada

**Rachel Flynn**

Canada

**Marie-Chantal Fortin**

Canada

**Maria Fusaro**

Italy

**Guillermo Garcia Garcia**

Mexico

**Amit Garg**

Canada

**Dianne Godkin**

Canada

**Lorraine Hamiwka**

Canada

**Brenda Hemmelgarn**

Canada

**Swapnil Hiremath**

Canada

**Michelle Hladunewich**

Canada

**Rachel Holden**

Canada

**Wilma Hopman**

Canada

**Shih-Han Huang**

Canada

**Ashley Irish**

Australia

**Matthew James**

Canada

**Min Jun**

Canada

**Joanne Kappel**

Canada

**Jolanta Karpinski**

Canada

**Mercedeh Kiaii**

Canada

**Bryce Kiberd**

Canada

**Scott Klarenbach**

Canada

**Greg Knoll**

Canada

**Paul Komenda**

Canada

**Ana Konvalinka**

Canada

**Ngan Lam**

Canada

**Norbert Lameire**

Belgium

**David Landry**

Canada

**Martine Leblanc**

Canada

**Jennifer MacRae**

Canada

**Braden Manns**

Canada

**Mark Marshall**

New Zealand

**Klemens Meyer**

USA

**Lisa Miller**

Canada

**Rudolf Obermayr**

Austria

**Donal O'Donoghue**

United Kingdom

**Masaki Ohsawa**

Japan

**Matthew Oliver**

Canada

**Suetonia Palmer**

New Zealand

**Neesh Pannu**

Canada

**Patrick Parfrey**

Canada

**Robert Pauly**

Canada

**Veronique Phan**

Canada

**Kevan Polkinghorne**

Australia

**Robert Richardson**

Canada

**Claudio Rigatto**

Canada

**Cassianne Robinson-Cohen**

USA

**Caren Rose**

Canada

**Susan Samuel**

Canada

**Kara Schick-Makaroff**

Canada

**Georg Schlieper**

Germany

**Stephen Seliger**

USA

**Rosalie Starzomski**

Canada

**Benedicte Stengel**

France

**Paul Stevens**

United Kingdom

**Zhaoli Sun**

USA

**Glen Taksler**

USA

**Navdeep Tangri**

Canada

**Karthik Tennankore**

Canada

**Mark Thomas**

United Kingdom

**Sheldon Tobe**

Canada

**Katrin Uhlig**

USA

**Ron Wald**

Canada

**James Wetmore**

USA

**Bryn Williams-Jones**

Canada

**Douglas Wilson**

Canada

**Karen Yeates**

Central African Republic

**Deborah Zimmerman**

Canada

